# Do NIR spectra collected from laboratory-reared mosquitoes differ from those collected from wild mosquitoes?

**DOI:** 10.1371/journal.pone.0198245

**Published:** 2018-05-31

**Authors:** Masabho P. Milali, Maggy T. Sikulu-Lord, Samson S. Kiware, Floyd E. Dowell, Richard J. Povinelli, George F. Corliss

**Affiliations:** 1 Department of Mathematics, Statistics and Computer Science, Marquette University, Wisconsin, United States of America; 2 Ifakara Health Institute, Environmental Health and Ecological Sciences Thematic Group, Ifakara, Tanzania; 3 Queensland Alliance of Agriculture and Food Innovation, The University of Queensland, Brisbane, Australia; 4 USDA, Agricultural Research Service, Center for Grain and Animal Health Research, Manhattan, KS, United States of America; 5 Department of Electrical and Computer Engineering, Marquette University, Wisconsin, United States of America; Universite Francois-Rabelais de Tours, FRANCE

## Abstract

**Background:**

Near infrared spectroscopy (NIRS) is a high throughput technique that measures absorbance of specific wavelengths of light by biological samples and uses this information to classify the age of lab-reared mosquitoes as younger or older than seven days with an average accuracy greater than 80%. For NIRS to estimate ages of wild mosquitoes, a sample of wild mosquitoes with known age in days would be required to train and test the model. Mark-release-recapture is the most reliable method to produce wild-caught mosquitoes of known age in days. However, it is logistically demanding, time inefficient, subject to low recapture rates, and raises ethical issues due to the release of mosquitoes. Using labels from Detinova dissection results in a mathematical model with poor accuracy. Alternatively, a model trained on spectra from laboratory-reared mosquitoes where age in days is known can be applied to estimate the age of wild mosquitoes, but this would be appropriate only if spectra collected from laboratory-reared and wild mosquitoes are similar.

**Methods and findings:**

We performed *k*-means (*k* = 2) cluster analysis on a mixture of spectra collected from lab-reared and wild *Anopheles arabiensis* to determine if there is any significant difference between these two groups. While controlling the numbers of mosquitoes included in the model at each age, we found two clusters with no significant difference in distribution of spectra collected from lab-reared and wild mosquitoes (*p* = 0.25). We repeated the analysis using hierarchical clustering, and similarly, no significant difference was observed (*p* = 0.13).

**Conclusion:**

We find no difference between spectra collected from laboratory-reared and wild mosquitoes of the same age and species. The results strengthen and support the on-going practice of applying the model trained on spectra collected from laboratory-reared mosquitoes, especially first-generation laboratory-reared mosquitoes.

## Introduction

The age of wild mosquitoes is commonly estimated by dissection of ovaries to determine their egg laying history [1–4]. Mosquitoes found to have laid eggs are assumed to be older than those without an egg laying history. While generally valid, this assumption can be wrong, as mosquitoes can be old without an egg laying history or young and have laid eggs. Dissection also is laborious, difficult, and limited to a few experts.

Near infrared spectroscopy (NIRS) is a high throughput technique that measures the chemical composition of biological samples [5–7]. NIRS has been applied to identify species of insects infecting stored grains [8]; to differentiate between species and subspecies of termites [9]; to age-grade houseflies [10], stored grain pests [11], and biting midges [12]; to estimate the age and identify species of morphologically indistinguishable laboratory reared and semi-field raised *Anopheles gambiae* and *Anopheles arabiensis* [13, 14]; to detect and identify two strains of *Wolbachia pipientis* (wMelPop and wMel) in male and female laboratory-reared *Aedes aegypti* [15]; and to classify the age of male and female wild-type and *Wolbachia*-infected *Aedes aegypti* [16].

Several studies report that NIRS can classify the age of lab-reared and semi-field mosquitoes into either less than or greater than seven days old with an accuracy exceeding 80% [13, 14, 17, 18]. Semi-field mosquitoes are offspring from wild caught females, raised within a large field cage (21x9.1x7.1m) that mimics the natural mosquito habitats [19]. The ability of NIRS to estimate the age of laboratory and semi-field raised mosquitoes is a prerequisite for accurately predicting the age of wild mosquito samples. However, it is challenging to develop or validate a NIRS model using a sample of wild mosquitoes, as it is difficult to obtain wild mosquitoes of a known age in days. As an alternative, models trained on spectra from laboratory-reared mosquitoes are applied to estimate the age of wild mosquitoes [16, 20], but no study has validated this generalization. Thus our objective is to determine if NIR spectra from laboratory-reared and wild mosquitoes are similar for the purposes of developing age-grading models.

Performing cluster analysis on the mixture of spectra collected from laboratory-reared and wild mosquitoes of the same species is one of the ways to address our objective. Cluster analysis is an unsupervised data partitioning process that groups a set of objects in such a way that objects in the same group (called a cluster) are more similar (in some way) to each other than to those in other groups (clusters) [21–25]. The term “unsupervised” means that during cluster analysis, no labels are given to the objects; clustering depends only on the set of features describing each object [24]. Ignoring labels from objects allows assigning of objects into groups using the objects’ features and not objects’ labels. For our problem, this means that during analysis we do not label spectra as laboratory or wild. We only provide entire spectra (absorbances) at 1851 wavelengths and partition the spectra into two groups depending only on their absorbances and not their labels (source of a mosquito or age). If spectra collected from lab-reared and those from wild are different, we expect them to be grouped into different clusters; otherwise they should distribute equally in the formed clusters. If lab-reared and wild mosquitoes produce similar spectra, the practice of applying models trained on lab-reared mosquitoes to estimate age of wild mosquitoes is appropriate.

In this study, we applied *k*-means [26] and hierarchical cluster analyses on a mixture of spectra collected from laboratory-reared and wild collected *An*. *arabiensis*. We tested the null hypothesis that there is no significant difference between the spectra collected from lab-reared and those from wild mosquitoes when other factors are equal.

## Materials and methods

### Ethics approval

Permission for blood feeding laboratory-reared mosquitoes and collecting wild mosquitoes from people’s homes was obtained from the Ifakara Health Institute (IHI) Review Board, under Ethical clearance No. IHRDC/EC4/CL.N96/2004 and No. IHI-IRB/No 17–2015, respectively. Oral consent was obtained from each adult volunteer involved in the study. The volunteers were given the right to refuse to participate or to withdraw from the experiment at any time.

### Mosquito collection

We used laboratory-reared *Anopheles arabiensis* mosquitoes of ages 1, 3, 5, 7, 9, 11, 15, 20, and 25 days post emergence with at least 80 mosquitoes in each age group, from the Ifakara Health Institute insectary. *An*. *arabiensis* mosquitoes were reared in 35cm x 35cm cages in a semi-field system [19] under ambient temperature and light-dark cycles. The humidity is artificially increased to approximately 80% during the dry season (May—October). Adult mosquitoes were daily given a 10% glucose solution and a blood meal twice per week via human arm (Ethical clearance No. IHRDC/EC4/CL.N96/2004). The insectary keeps records of mosquitoes from egg laying to adult emergence, and the cages are labeled so that mosquito ages are easily identified.

Wild *An*. *arabiensis* mosquitoes were collected using CDC light traps [27] in Minepa, a village in south-eastern Tanzania. The traps were set in selected houses in the evening and collected the next morning (Ethical clearance No. IHI-IRB/No 17–2015). Live *Anopheles gambiae* complex mosquitoes were sorted from other mosquitoes from the traps and put in a small cage and provided with 10% sugar solution. The sorted *Anopheles gambiae* complex mosquitoes were transported to the Ifakara Health Institute laboratory for spectra collection.

### Spectra collection

Before scanning, both laboratory-reared and wild mosquitoes were killed by freezing for 20 minutes. Spectra were collected using a LabSpec 5000 NIR spectrometer (ASD Inc, Longmont, Colorado) and pre-processed as previously described [13]. After scanning, wild mosquitoes were dissected to determine their egg laying history, followed by polymerase chain reaction (PCR) to identify species type [28]. Only spectra from wild mosquitoes identified as *Anopheles arabiensis* were used for analysis. Our final dataset contained spectra from 863 laboratory-reared mosquitoes and 927 wild-caught mosquitoes at wavelengths 500–2350 nm.

### Clustering analysis

After spectra pre-processing, we ignored associated labels identifying the source of mosquitoes (laboratory or wild) and performed cluster analysis in three different approaches using *k*-means and hierarchical clustering methods [22, 26].

#### Clustering approach one

We mixed all 863 spectra collected from laboratory-reared *An*. *arabiensis* and all 927 spectra collected from wild *An*. *arabiensis* and performed *k*-means cluster analysis on the entire data set (using 1851 absorbances at wavelengths between 500–2350 nm) in Matlab. *K*-means cluster analysis, also known as Lloyd's algorithm [26], starts by arbitrarily choosing cluster centers known as centroids, depending on the number of clusters needed. In our case, we needed two clusters to determine if there is any significant difference between spectra collected from laboratory-reared and wild mosquitoes, so the number of centroids is two. The next step was to compute distances from each mosquito (spectrum) to each centroid and assign each mosquito to its closest centroid. There are different ways to compute distance, but this study used squared Euclidean distance [29]. Finally, the average distance of mosquitoes assigned to each centroid was computed. The process was repeated by selecting new centroids and reassigning mosquitoes until the average distance to the centroids was minimized.

After the clusters were formed, the next step was to evaluate their quality by computing the silhouette coefficient (SC) of the cluster [30–34]. SC is defined as the measure of how objects in the same cluster are similar and different from the objects in the other clusters [21, 35]. SC of the cluster is an average of all SC of objects in that cluster, computed using Eq 1.

Let

*s(o)* = Silhouette coefficient of a single object *‘o’*
*a(o)* = Average distance of object *‘o’* to the other objects in its cluster
*b(o)* = Average distance of object *‘o’* to other objects in the nearest cluster.

Then

$$s(o)=\frac{\left[b(o)-a(o)\right]}{max(a(o),\;b(o))}$$ (1)

The lower the ‘*a’* value the better, and the higher the *‘b’* value the better.

SC values ranges from -1 to +1, where +1 indicates that an object is well matched to objects in its own cluster and poorly matched to objects in neighboring clusters [21]. If most objects in the cluster have high SC, then the clustering is appropriate; otherwise, (lower SC) the clustering is inappropriate. Since SC of -1 and +1 are extreme values, the interpretation of high or low for SC values between– 1 and +1 can be subjective. The interpretation of SC as reported by Struyf et al. [23] is summarized in Table 1 and often is used by studies [36–40] involving cluster analysis.

**Table 1:** Interpretation of the silhouette values for partitioning methods.

Silhouette coefficient	Proposed interpretation
**0.71–1.00**	A strong cluster has been found
**0.51–0.70**	A reasonable cluster has been found
**0.26–0.50**	The cluster is weak and could be artificial
**≤ 0.25**	No substantial cluster has been found

We repeated the analysis using hierarchical clustering. Hierarchical clustering groups data objects into a hierarchy or tree of clusters [41]. Hierarchical clustering often is believed to form higher quality clusters than *k*-means, but it is limited because of its quadratic time complexity [42]. An advantage of using *k*-means is that its time complexity is linear in the number of objects, but it is thought to produce lower quality clusters [42]. Applying both *k*-means and hierarchical approaches takes advantage of the strengths in both methods. In addition to forming quality clusters, hierarchical clustering iteratively builds different levels of clusters from clusters consisting of individual objects to one large cluster, providing a platform to analyze in detail how mosquitoes distribute in different levels of clusters in the hierarchy.

We built the hierarchical tree using an agglomerative method (bottom-up strategy) [41]. The agglomerative method starts by treating individual mosquitoes as clusters and then iteratively merges them into larger clusters based on their similarities [41]. When generating a tree, we restricted the number of leaf nodes to thirty for both simplicity of viewing the tree and analysis of how mosquitoes distribute from higher to lower level clusters.

#### Clustering approach two

Several studies [13, 14, 17, 18] show that spectra can be used to classify mosquitoes into two age classes (less than seven days against greater or equal to seven days old), implying that age of a mosquito should not be ignored as a factor contributing the formation of two clusters. In addition, the age structure of wild mosquito populations generally follows an exponential decay curve (where a constant proportion of mosquitoes die each day) [1, 4, 43–46]. If our wild mosquito data have such an age distribution, and since the laboratory-reared mosquitoes have a uniform age distribution by experimental design, there is high chance that clustering using our first approach is influenced by this age structure difference between the two data sets (wild and laboratory-reared mosquitoes). Hence, in our second approach, we explore possible age-dependencies that may influence our clustering. We repeated the *k*-means and hierarchical analyses, this time controlling the number of mosquitoes per age in the dataset. Lacking age in days labels for spectra collected from wild mosquitoes, we controlled the number of mosquitoes per age in three different ways.

**Fig 1:**
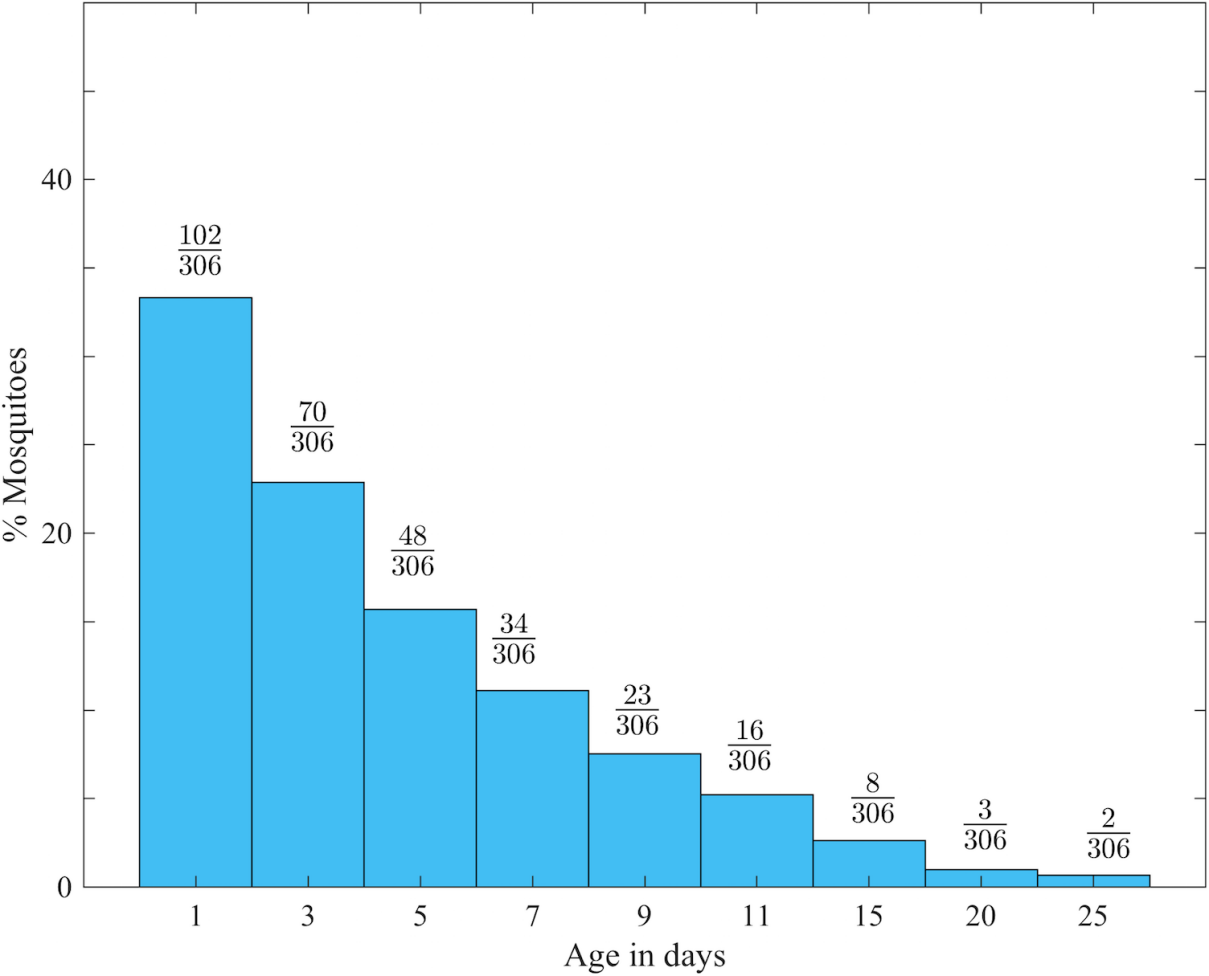
Number of laboratory-reared mosquitoes per age, selected to simulate the age distribution of wild mosquitoes.

First, we transformed the initial uniform age structure of laboratory-reared mosquitoes to fit the published age structure (exponential decay curve) of wild mosquito populations [1, 4, 43–46]. We simulated the population of laboratory-reared mosquitoes with 102 one-day-old mosquitoes (based on the number of one day old in the data set) and computed the composition of other ages in the population using a published daily survival rate of 0.83 [46]. The computed number of laboratory-reared mosquitoes with ages other than one day old required to form an exponential decay distribution was randomly selected from a stratified-by-age original laboratory-reared mosquito data set. There are a number of assumptions when simulating the exponential age distribution of mosquitoes [46, 47]. The main assumptions for this simulation are: no addition of other mosquitoes into the population; the probability of a mosquito surviving one day is constant in all age classes. This process yielded a total of 306 laboratory-reared mosquitoes in an imitated population. More on how to simulate the age structure of wild mosquito populations can be found at [44, 46, 47]. Fig 1 presents the age composition in a population of laboratory-reared mosquitoes selected to imitate an exponential age decay curve.

We then randomly selected 306 spectra collected from wild mosquitoes to match the number of laboratory-reared mosquitoes in the selected population, mixed the two populations (selected laboratory-reared mosquitoes to form an exponential decay distribution and randomly selected wild mosquitoes), and repeat *k*-means and hierarchical cluster analyses, respectively, as in approach one.

**Fig 2:**
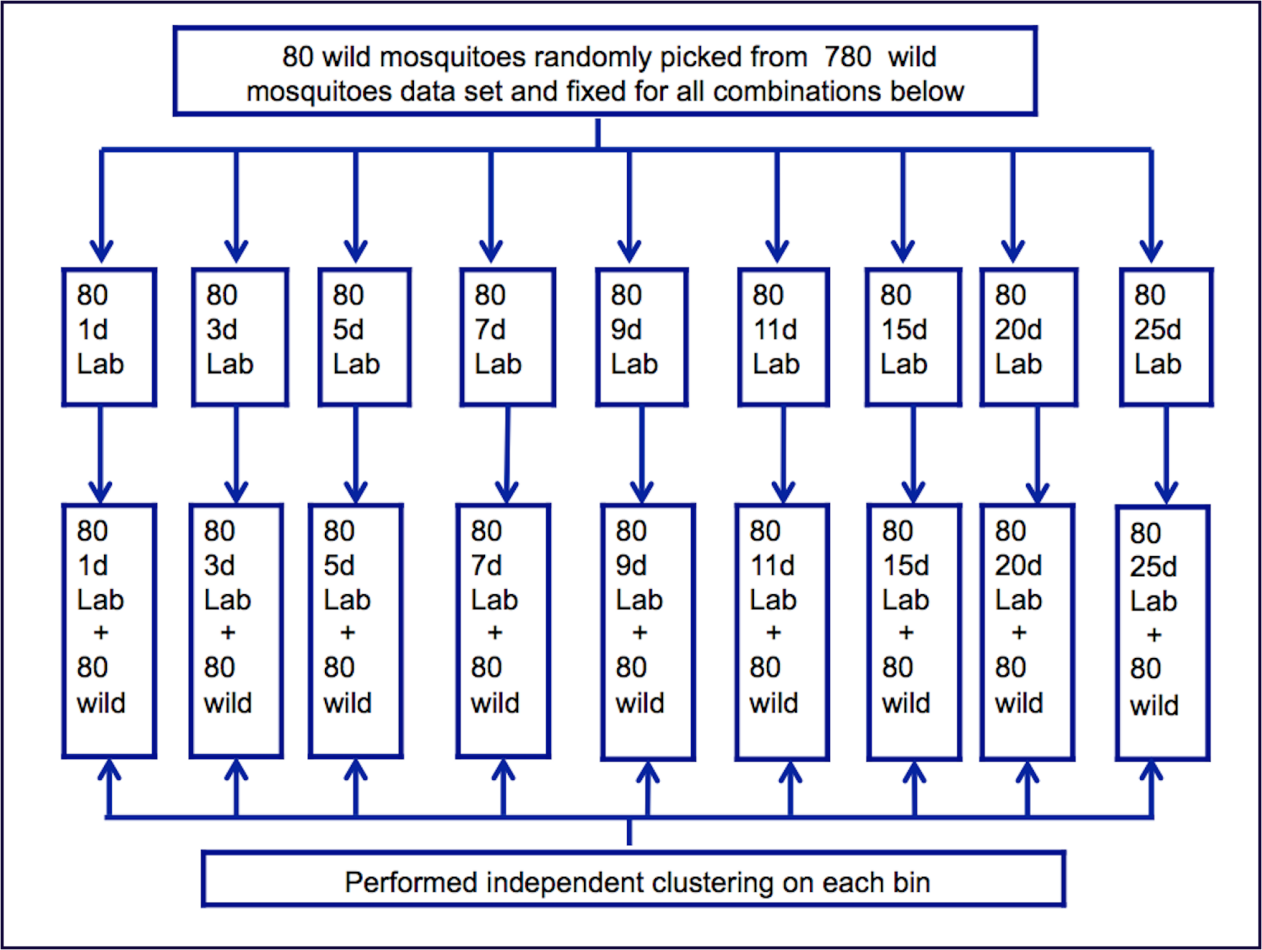
Illustration of the second method used to control number of mosquitoes per age during clustering approach two.

Second, we randomly selected 80 spectra collected from wild mosquitoes and maintained them for the rest of the analysis, while changing the age of the laboratory-reared mosquitoes. We mixed 80 spectra of one-day-old laboratory-reared *An*. *arabiensis* and 80 randomly selected spectra from wild *An*. *arabiensis* and performed the *k*-means analysis as in the first approach. We repeated the process for the remaining ages (i.e., 3, 5, 7, 9, 11, 15, 20, and 25) of laboratory-reared mosquitoes, while keeping the spectra from wild *An*. *arabiensis* unchanged (same 80 randomly selected). Fig 2 illustrates the process.

Third, based on the results represented in Table 2, yielded by the method illustrated in Fig 2, only laboratory-reared mosquitoes that were 3, 5, and 25 days old clustered differently from the randomly selected sample of wild mosquitoes. We hypothesized that the wild mosquitoes in the data set could have been newly emerged but not too old, causing few or none of them to be 3, 5, or 25 days old. Hence, creating age structure differences between laboratory reared and wild mosquito populations used in the first approach. Therefore, spectra associated to mosquitoes that are 3, 5, and 25 days old were excluded from the laboratory-reared data set to determine if they influenced clusters formation in the first approach. We retained the 598 spectra associated with 1, 7, 9, 11, 15, and 20-day old laboratory-reared mosquitoes. We mixed them with all 927 wild spectra and performed the analysis as in the first approach.

**Table 2:** Number of mosquitoes in clusters when 80 spectra collected from wild mosquitoes were randomly selected and maintained for the rest of the analysis, while changing the age of the laboratory-reared mosquitoes.

Age	Cluster	Number of laboratory	Number of wild	Total	Av. SC*	χ2**	*p*-value
**1**	1	34	38	72	0.48		
	2	46	42	88	0.77	0.40	0.53
	Total	80	80	160			
**3**	1	33	46	79	0.64		
	2	47	34	81	0.65	4.23	0.04
	Total	80	80	160			
**5**	1	46	29	75	0.69		
	2	34	51	85	0.69	7.31	0.01
	Total	80	80	160			
**7**	1	47	38	85	0.67		
	2	33	42	75	0.73	2.03	0.15
	Total	80	80	160			
**9**	1	37	42	79	0.71		
	2	43	38	81	0.66	0.63	0.43
	Total	80	80	160			
**11**	1	30	40	70	0.82		
	2	50	40	90	0.41	2.54	0.11
	Total	80	80	160			
**15**	1	34	43	77	0.45		
	2	46	37	83	0.78	2.03	0.15
	Total	80	80	160			
**20**	1	35	42	77	0.60		
	2	45	38	83	0.74	1.23	0.27
	Total	80	80	160			
**25**	1	47	29	76	0.66		
	2	33	51	84	0.70	8.12	0.01
	Total	80	80	160			

* Average silhouette coefficient

**Chi square

We did not use age classification labels from ovary dissection to control the number of wild mosquitoes per age because the ovary dissection method only determines the physiological age of mosquitoes and cannot infer mosquito age in days [1–4]. The method classifies mosquitoes as relatively young (not laid eggs) or old (laid eggs) based on egg laying status. This classification can be misleading, as mosquitoes lay eggs after getting blood for egg development. Therefore, a mosquito can be old without a gonotrophic history or young and have laid eggs.

#### Clustering approach three

Since the current NIRS results were achieved by training a model on six to ten components using partial least squares regression (PLSR), we performed PLSR on the spectra to reduce spectra features from 1851 absorbances to ten components and repeated *k*-means cluster analysis as in the first approach. Feature reduction using PLSR reduces noise in data without losing important information. PLSR reduces features by finding components associated with all features (absorbances) while considering dependent variables (laboratory or wild in our case) [48, 49].

## Results

We find no difference in spectra collected from laboratory-reared and wild mosquitoes when the number of mosquitoes per age between two groups of mosquitoes is controlled.

### Clustering approach one

**Fig 3:**
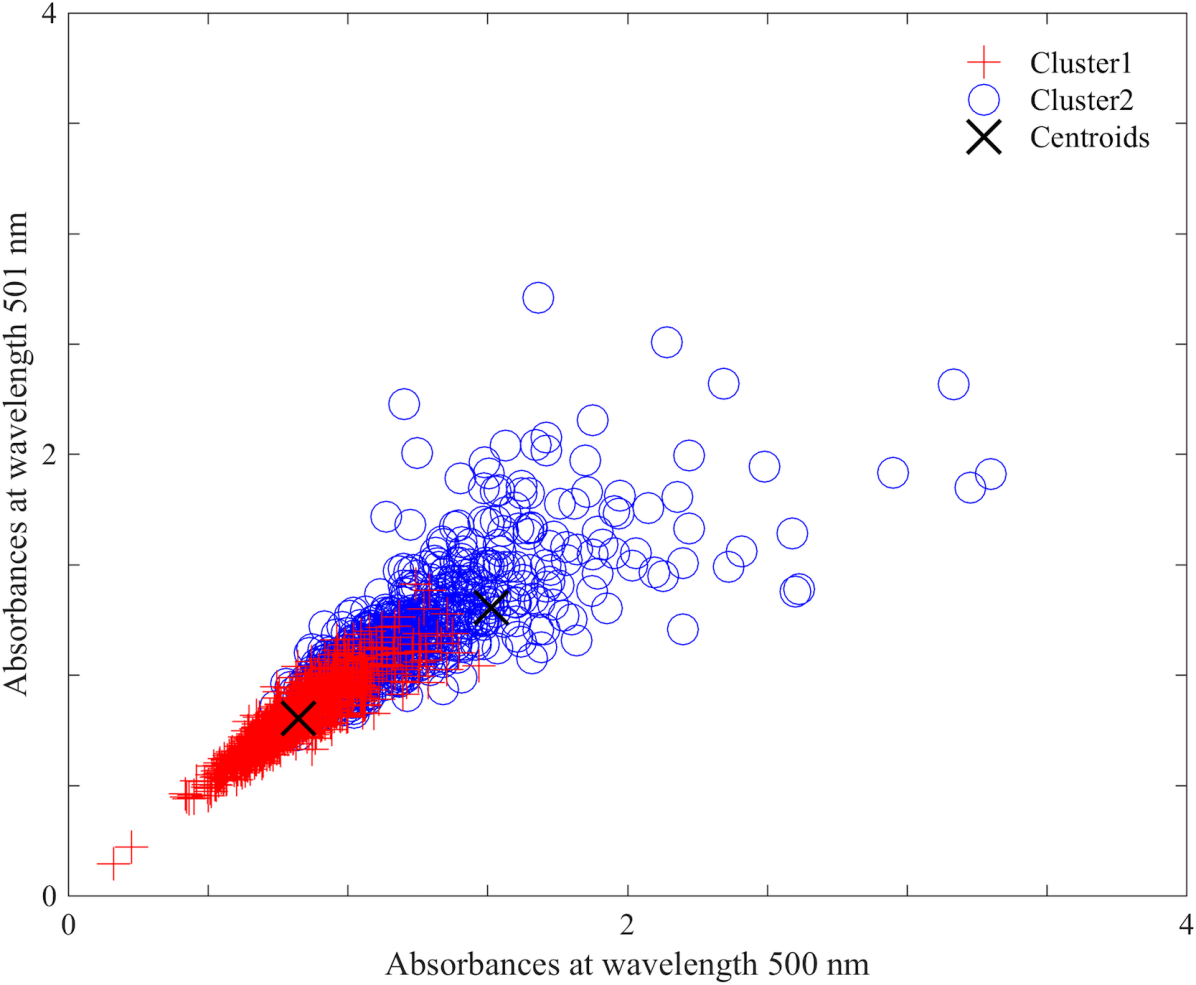
Two-dimensional plot of clusters using absorbances at 500 nm and 501 nm, when number of mosquitoes per age was not controlled.

Due to the multidimensional nature of the formed clusters after *k*-means analysis, it is not possible to represent the formed cluster with all absorbances in the spectra in two dimensions. Instead, for illustrative purposes, Fig 3 represents the formed clusters plotted using spectra according to their absorbance at two different wavelengths, 500 and 501 nm (these two absorbances at 500 nm and 501 nm should not be confused as the only absorbances used for clustering, we used all the absorbances in the spectra during cluster analysis). Similar displays were generated using absorbances at different wavelengths, and the patterns of the displays were similar. Fig 3 shows that there are two clusters, despite some overlapping of spectra (objects) in both clusters.

**Fig 4:**
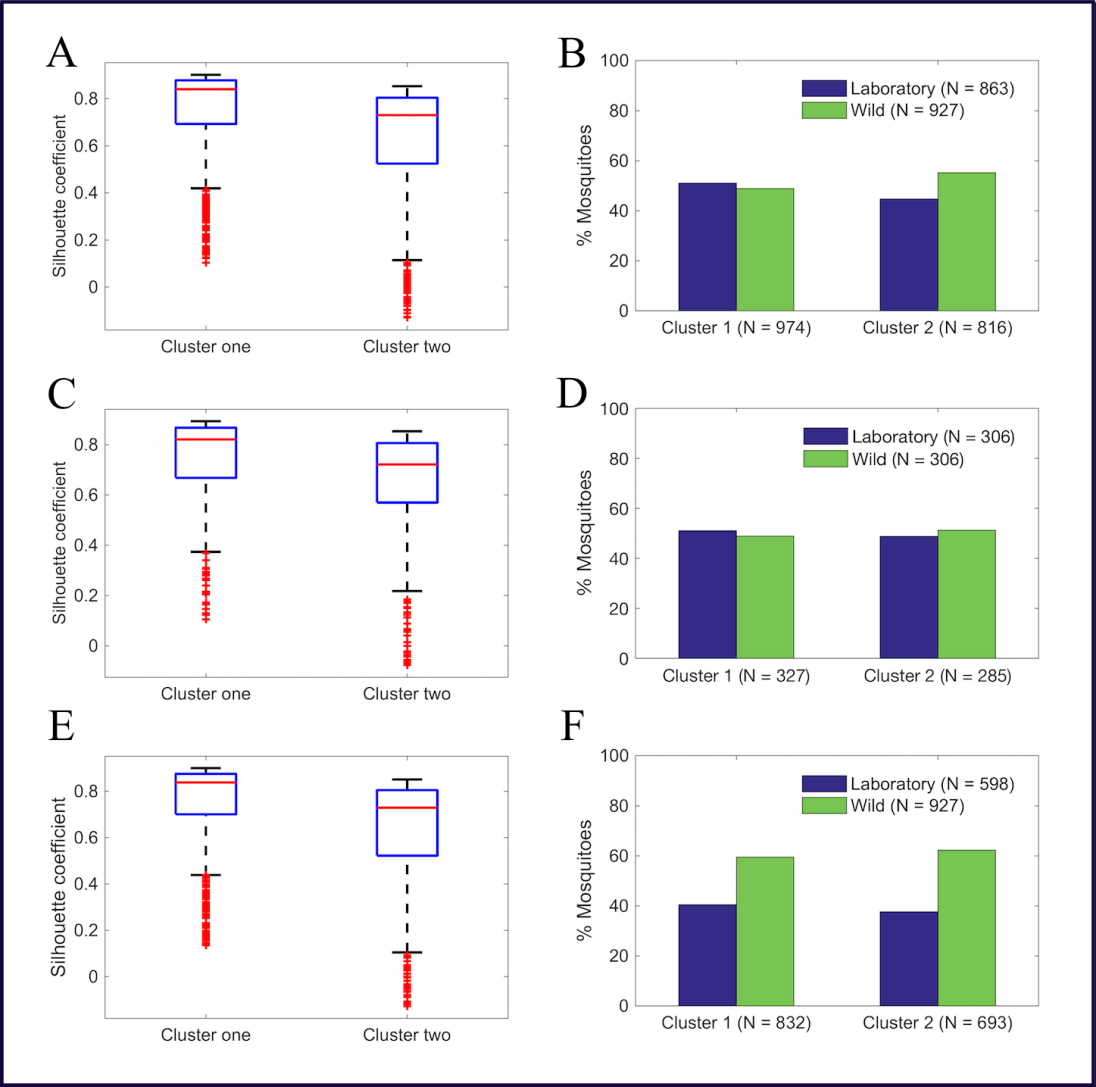
Box plots of silhouette coefficients and bar graphs of percentage of mosquitoes, respectively, showing the quality and distribution of laboratory-reared and wild mosquitoes in clusters after *k*-means analysis. A and B, number of mosquitoes per age was not controlled (*p* = 0.01), C and D, age structure of laboratory-reared mosquitoes was standardized to match the published age structure of wild mosquitoes (*p* = 0.57), E and F, laboratory-reared mosquitoes at 3, 5, and 25-day old were not included in the analysis (*p* = 0.26). *P* stands for p value and N for the number of mosquitoes.

Using Eq 1, the qualities of the two formed mosquito clusters were evaluated and scored mean SC of 0.63 and 0.75 for clusters one and two, respectively. Fig 4A represents a box plots providing more detailed information (minimum, 25^th^ percentile, median, 75^th^ percentile and maximum) on the SC of mosquitoes in the formed clusters. By the SC interpretation in Table 1, the clusters shown in Fig 3 are reasonable and strong, respectively.

After finding the quality of the formed clusters to be reasonable and strong, a contingency table was generated, and a χ2 statistical test was performed to determine if there is a significant difference in distribution of laboratory-reared and wild mosquitoes in the two clusters. That is, do the two clusters capture the sources of the mosquitoes? Fig 4B and S1 Table in the supporting information present the results, showing a significant difference (*p* = 0.01) in the distribution of both laboratory-reared and wild mosquitoes in the clusters. Cluster one has more laboratory-reared mosquitoes, while cluster two has more wild mosquitoes.

**Fig 5:**
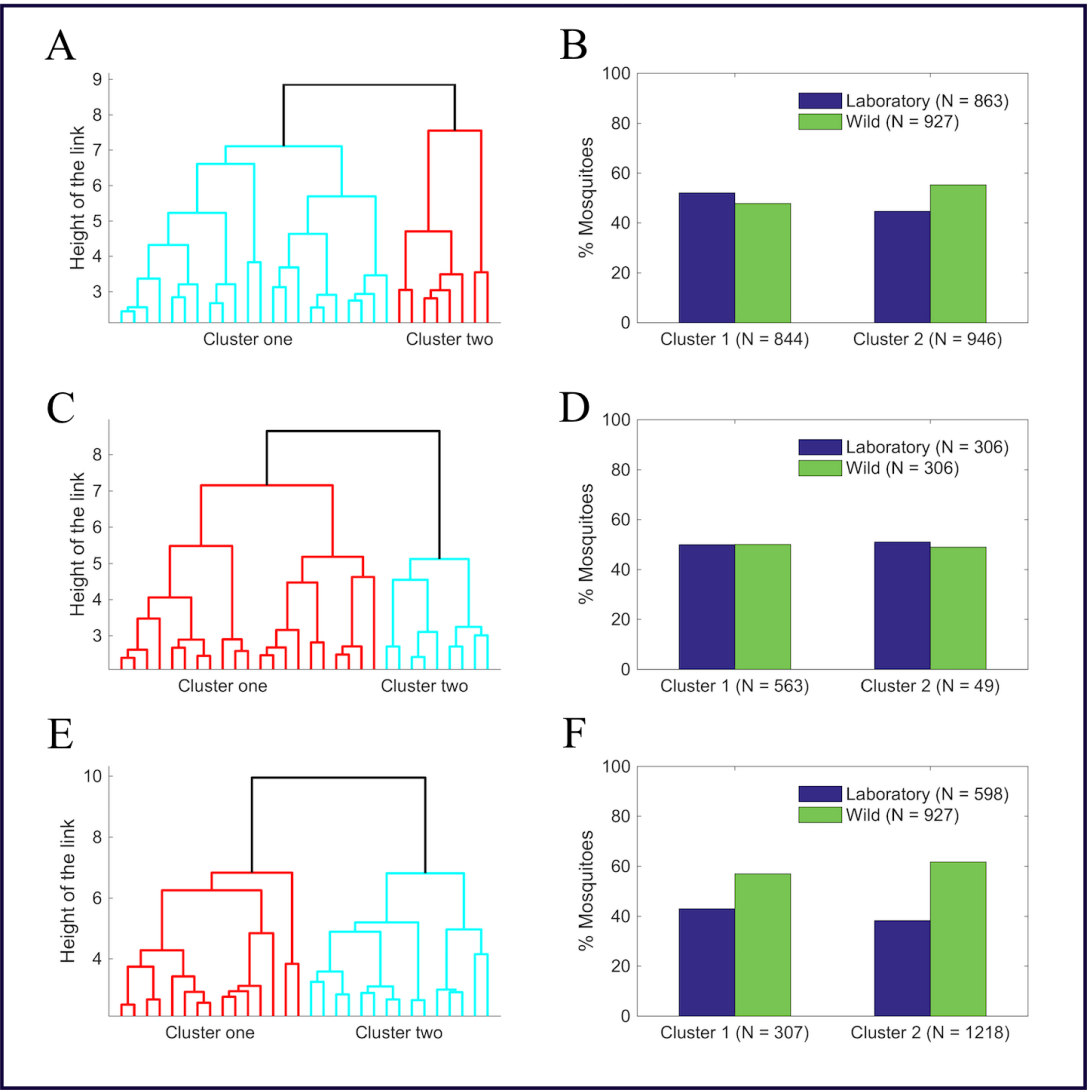
Hierarchical tree and bar graphs showing distributions of laboratory-reared and wild mosquitoes in clusters formed by hierarchical cluster analysis. A and B, number of mosquitoes per age was not controlled (*p* < 0.01); C and D, the age structure of laboratory-reared mosquitoes was fit to an exponential decay distribution to match the published age structure of wild mosquitoes (*p* = 0.76); and E and F, laboratory-reared mosquitoes at 3, 5, and 25-day old were not included in the analysis (*p* = 0.13).

Fig 5A and 5B (also S1 Table in the supporting information), respectively, present the hierarchical tree and the bar graph generated after hierarchical clustering, showing formed clusters with more laboratory-reared mosquitoes in cluster one and more wild mosquitoes in cluster two. The chi-square test found the difference to be significant (*p* < 0.01), which agrees with the results of *k*-means.

S2 Table in the supporting information presents the distributions of laboratory and wild mosquitoes in each of the thirty nodes showing almost all nodes containing both types of mosquitoes. Having both laboratory and wild mosquitoes in most of the formed clusters (node) at the level of thirty clusters (nodes) strongly suggest that the source of mosquitoes was not the criterion used in forming two clusters.

### Clustering approach two

First, the formed clusters after *k*-means analysis on the dataset with number of mosquitoes per age controlled using an exponential decay curve scored SC of 0.74 and 0.64, showing the cluster qualities to be strong and reasonable, respectively (Fig 4C). The distribution of mosquitoes in the clusters was independent of the source of mosquitoes (Fig 4D and S1 Table in the supporting information). When we repeated hierarchical clustering, a hierarchical tree (Fig 5C) with no significant difference (*p* = 0.88) in the distribution of laboratory-reared and wild mosquitoes between the two-formed clusters (Fig 5D and S1 Table) was generated. S2 Table represents mosquito distributions in each of the thirty nodes still showing most of the nodes consisting of both laboratory-reared and wild mosquitoes, further suggesting that clustering is independent of the source of mosquitoes. The outcome strengthens our hypothesis that age influenced the previous clustering.

Second, following *k*-means analysis on spectra with the number of mosquitoes per age controlled as illustrated in Fig 2, the source of mosquitoes influenced the formation of clusters when clustering involved laboratory-reared mosquitoes at ages 3, 5, and 25 days old (Table 2). For the remaining age groups, clustering was independent of the source of mosquitoes. The likely explanation for these results is that a majority of the wild mosquitoes collected could have been newly emerged but not too old.

Third, Fig 4E represents the silhouette coefficients of mosquitoes in each formed cluster when *k*-means analysis was performed on the dataset with the number of mosquitoes per age controlled by excluding spectra associated with 3, 5, and 25-day old laboratory-reared mosquitoes from the original dataset. The figure shows the quality of clusters was not compromised with the removal of 3, 5, and 25-day old laboratory-reared mosquitoes in the analysis. Fig 4F and S1 Table, in the supporting information represent the results from *k*-means analysis showing no significant difference between spectra collected from laboratory-reared and wild *An*. *arabiensis* (*p* = 0.26). Fig 5E represents a hierarchical tree generated after hierarchical clustering was performed on the same dataset (number of mosquitoes per age controlled by removing spectra associated with 3, 5, and 25-day old laboratory-reared mosquitoes from the original dataset) showing no significant difference (*p* = 0.13) in the distribution of laboratory-reared and wild *An*.*arabiensis* between clusters (Fig 5F and S1 Table in the supporting information). S2 Table presents mosquito distributions in each of the thirty nodes, showing the same trend of each node consisting both laboratory-reared and wild *An*. *arabiensis*. These results strongly suggest that the results from clustering approach one were influenced by mosquito age differences and not their source.

### Clustering approach three

After performing *k*-means clustering on spectra with their features reduced from 1851 absorbances to ten PLS components, we found no substantial clusters with SC below 0.25 (S1A and S1B Fig in the supporting information). The results strengthen the findings obtained when the number of mosquitoes per age was controlled, where it was found that no difference between spectra collected from lab-reared and wild mosquitoes of the same species. The results further suggest that clustering in the first approach was influenced by age.

## Discussion

In this study, we investigated whether there is any significant difference between NIR spectra collected from laboratory-reared and wild mosquitoes. Our results show that *k*-means and hierarchical cluster analyses on the mixture of spectra without controlling the number of mosquitoes per age produced clusters associated with the source of the spectra. This could infer that there is a difference between spectra collected from laboratory-reared mosquitoes and those collected from the wild. However, different factors apart from the source of the spectra may have contributed to the results. Age of a mosquito is one of the most important factors to consider, as different studies [13, 14, 17] have already shown that spectra can be used to estimate the ages of mosquitoes, implying that mosquitoes of the same species but different ages can be differentiated using spectra. Hence, clustering of spectra can occur based on age differences of mosquitoes. Physiological status (laid eggs or not, blood fed or not) of a mosquito also can influence the cluster formation. Ntamatungiro et al. [18] showed there is an influence of physiological status of a mosquito on the spectra.

Therefore, we explored whether the age of mosquitoes might be influencing the results in the first approach. We repeated *k*-means and hierarchical cluster analyses on the mixture of spectra, while controlling the number of mosquitoes per age in the dataset. The results showed no influence of the source of mosquitoes on forming clusters. This means in the first approach, age played an important role in cluster formation. When we performed cluster analysis while controlling the egg laying status (as one way to determine the influence of physiological status) of both wild and laboratory-reared mosquitoes, results showed no influence on cluster formation.

Since partial least squares analysis has been shown to be effective for age-classification of lab-reared mosquitoes, we performed partial least square analysis on the spectra to reduce the number of features before we did cluster analysis. Feature reduction using PLSR can help during analysis by reducing noise in data without losing important information. Initially, the spectra had 1851 features, which can introduce errors during cluster analysis. PLSR discards only a little information when reducing features; instead it finds components associated with all features while considering dependent variables [48, 49]. When we applied PLSR and performed *k*-means clustering on the reduced features (ten components), we found very poor clustering, with average SCs below 0.21, which indicates that there is no clustering tendency in the data [21, 23]. These results strengthened the results obtained when the age of laboratory-reared mosquitoes was controlled.

## Conclusions

Having two clustering methods with different clustering mechanisms reaching the same conclusion, we fail to reject the null hypothesis that there is no significant difference between the spectra collected from laboratory-reared and those from wild mosquitoes of the same age and species. Thus, our study finds that there is no difference between NIR spectra collected from laboratory-reared and wild collected mosquitoes of the same species when number of mosquitoes per age is controlled. While further studies may be required to explore a more appropriate way to estimate age of wild mosquitoes, these results strengthen the ongoing practice of training models to estimate age of wild mosquitoes using spectra collected from laboratory-reared mosquitoes [16, 20]. Although model estimates have limitations [50, 51, 52, 53], they allow us to make inferences in situations where it is impractical to determine the ground truth, such as the actual age of wild-caught mosquitoes. While the practice of applying a model trained on first generation laboratory-reared mosquitoes to estimate wild mosquitoes is not ideal, the results from this study support the practice. We show that this practice is likely reliable enough to give insight into the age structure of a wild mosquito population, especially when complemented with other existing knowledge on age structure of wild mosquitoes.

## Supporting information

S1 Appendix Excel file with the data used in the analysis. Column header, wavelengths in ‘nm’.
(XLSX)

S2 Appendix Matlab code used to run the analysis. (M)

S1 Fig Two-dimensional plot of clusters using first and second PLS components (A), and box plots, showing the silhouette coefficient of each spectrum (object) in its associated cluster (B) when partial least squares was applied to reduce the data dimension before clustering.
(TIF)

S1 Table **Number and type of mosquitoes in clusters when k-means and hierarchical clustering were applied to spectra with:** Age of mosquitoes not controlled (A_k_ and A_h_, respectively); Age structure of laboratory-reared mosquitoes controlled to match the published age structure of wild mosquitoes (B_k_ and B_h,_ respectively) and; Laboratory-reared mosquitoes at age 3, 5, and 25-day old not included in the analysis (C_k_ and C_h,_ respectively). *X*^*2*^ = computed chi-square.
(DOCX)

S2 Table **Number and type of mosquitoes in leaf nodes of the hierarchical tree:** A) Age of mosquitoes not controlled; B) Age of mosquitoes controlled by selecting age of laboratory-reared mosquitoes to fit the published age distribution of wild mosquitoes; C) Laboratory-reared mosquitoes at age 3, 5, and 25-day old excluded in the analysis.
(DOCX)
