# Supplementary figures and images for: Do NIR spectra collected from laboratory-reared mosquitoes differ from those collected from wild mosquitoes?
Source: PLoS One. 2018 May 31;13(5):e0198245. doi: 10.1371/journal.pone.0198245 (PMC5978888; doi:10.1371/journal.pone.0198245)

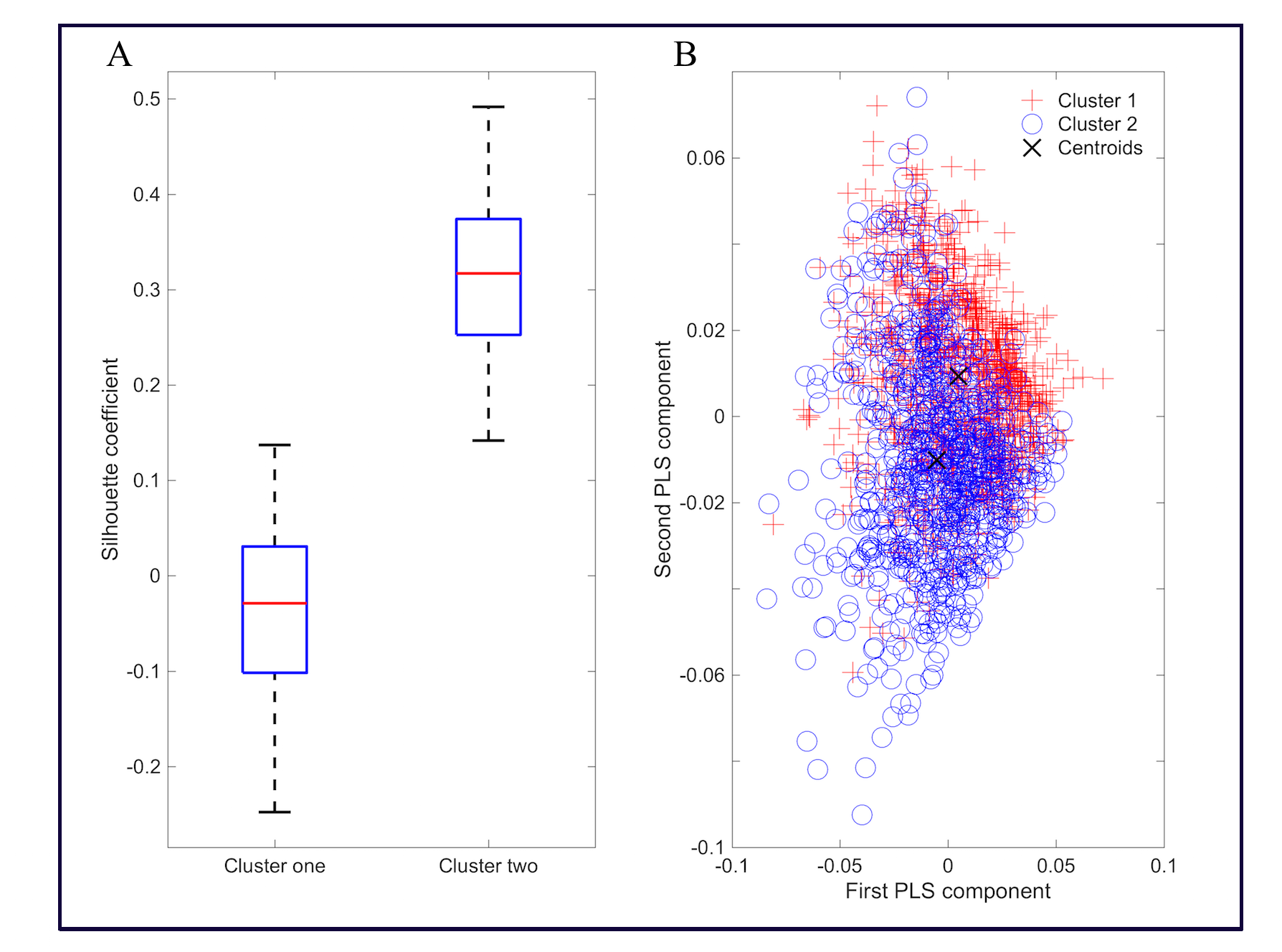

Supplement: S1 Fig — Two-dimensional plot of clusters using first and second PLS components (A), and box plots, showing the silhouette coefficient of each spectrum (object) in its associated cluster (B) when partial least squares was applied to reduce the data dimension before clustering. (TIF) [file pone.0198245.s003.tif]
