# Supplementary material for: Do NIR spectra collected from laboratory-reared mosquitoes differ from those collected from wild mosquitoes?
Source: PLoS One. 2018 May 31;13(5):e0198245. doi: 10.1371/journal.pone.0198245 (PMC5978888; doi:10.1371/journal.pone.0198245)
Supplement: S1 Table — Number and type of mosquitoes in clusters when k-means and hierarchical clustering were applied to spectra with: Age of mosquitoes not controlled (Ak and Ah, respectively); Age structure of laboratory-reared mosquitoes controlled to match the published age structure of wild mosquitoes (Bk and Bh, respectively) and; Laboratory-reared mosquitoes at age 3, 5, and 25-day old not included in the analysis (Ck and Ch, respectively). X2 = computed chi-square. (DOCX) [file pone.0198245.s004.docx]

| Spectra  data | Clustering  technique | Cluster | Number of Laboratory  mosquitoes | Number of Wild  mosquitoes | Total | *X*^2^ | *P*-value |
| --- | --- | --- | --- | --- | --- | --- | --- |
| A_k_ | K-means | 1 | 498 | 476 | 974 | 7.28 | 0.01 |
|  |  | 2 | 365 | 451 | 816 |  |  |
|  |  | Total | 863 | 927 | 1790 |  |  |
| A_h_ | Hierarchical | 1 | 365 | 479 | 844 | 15.78 | < 0.01 |
|  |  | 2 | 498 | 448 | 946 |  |  |
|  |  | Total | 863 | 927 | 1790 |  |  |
| B_k_ | K-means | 1 | 167 | 160 | 327 | 0.32 | 0.57 |
|  |  | 2 | 139 | 146 | 285 |  |  |
|  |  | Total | 306 | 306 | 612 |  |  |
| B_h_ | Hierarchical | 1 | 58 | 61 | 119 | 0.094 | 0.76 |
|  |  | 2 | 248 | 245 | 493 |  |  |
|  |  | Total | 306 | 306 | 612 |  |  |
| C_k_ | K-means | 1 | 337 | 495 | 832 | 1.28 | 0.26 |
|  |  | 2 | 261 | 432 | 693 |  |  |
|  |  | Total | 598 | 927 | 1525 |  |  |
| C_h_ | Hierarchical | 1 | 132 | 175 | 307 | 2.31 | 0.13 |
|  |  | 2 | 466 | 752 | 1218 |  |  |
|  |  | Total | 598 | 927 | 1525 |  |  |
