# Supplementary material for: Do NIR spectra collected from laboratory-reared mosquitoes differ from those collected from wild mosquitoes?
Source: PLoS One. 2018 May 31;13(5):e0198245. doi: 10.1371/journal.pone.0198245 (PMC5978888; doi:10.1371/journal.pone.0198245)
Supplement: S2 Table — Number and type of mosquitoes in leaf nodes of the hierarchical tree: A) Age of mosquitoes not controlled; B) Age of mosquitoes controlled by selecting age of laboratory-reared mosquitoes to fit the published age distribution of wild mosquitoes; C) Laboratory-reared mosquitoes at age 3, 5, and 25-day old excluded in the analysis. (DOCX) [file pone.0198245.s005.docx]

|  | **A** | | **B** | | **C** | |
| --- | --- | --- | --- | --- | --- | --- |
| Leaf  node | No. Lab  mosquitoes | No. Wild  mosquitoes | No. Lab  mosquitoes | No. Wild mosquitoes | No. Lab  mosquitoes | No. Wild  mosquitoes |
| 1 | 93 | 42 | 3 | 5 | 114 | 74 |
| 2 | 92 | 151 | 1 | 5 | 64 | 70 |
| 3 | 47 | 10 | 34 | 18 | 90 | 77 |
| 4 | 37 | 89 | 11 | 23 | 28 | 88 |
| 5 | 10 | 32 | 26 | 17 | 20 | 18 |
| 6 | 158 | 69 | 20 | 3 | 1 | 9 |
| 7 | 1 | 5 | 15 | 28 | 0 | 3 |
| 8 | 19 | 23 | 39 | 26 | 6 | 12 |
| 9 | 42 | 101 | 2 | 5 | 6 | 34 |
| 10 | 27 | 78 | 18 | 11 | 0 | 10 |
| 11 | 7 | 12 | 15 | 25 | 55 | 43 |
| 12 | 110 | 68 | 4 | 5 | 1 | 20 |
| 13 | 0 | 2 | 9 | 7 | 6 | 9 |
| 14 | 2 | 9 | 0 | 1 | 0 | 3 |
| 15 | 20 | 38 | 25 | 25 | 0 | 2 |
| 16 | 8 | 7 | 7 | 4 | 1 | 0 |
| 17 | 6 | 9 | 3 | 1 | 24 | 14 |
| 18 | 13 | 21 | 8 | 10 | 82 | 194 |
| 19 | 1 | 4 | 0 | 4 | 49 | 71 |
| 20 | 0 | 2 | 19 | 13 | 24 | 87 |
| 21 | 3 | 6 | 23 | 31 | 9 | 36 |
| 22 | 0 | 2 | 1 | 3 | 6 | 15 |
| 23 | 0 | 1 | 20 | 24 | 8 | 20 |
| 24 | 48 | 32 | 0 | 1 | 3 | 6 |
| 25 | 8 | 32 | 0 | 1 | 1 | 4 |
| 26 | 0 | 1 | 0 | 2 | 0 | 2 |
| 27 | 111 | 73 | 2 | 5 | 0 | 1 |
| 28 | 0 | 3 | 0 | 1 | 0 | 3 |
| 29 | 0 | 3 | 0 | 1 | 0 | 1 |
| 30 | 0 | 2 | 1 | 1 | 0 | 1 |
